# Universal Factors Affecting Emergency Department Crowding

**DOI:** 10.5812/atr.9835

**Published:** 2013-02-01

**Authors:** Arvind Venkat

**Affiliations:** 1 Department of Emergency Medicine , Allegheny General Hospital, Temple University School of Medicine and Drexel University College of Medicine, Pittsburgh, USA

**Keywords:** Crowding, Emergency Medicine, Emergency Department Operation

*Dear Editor,*

I read with great interest the article entitled “Application of Queuing Analytic Theory to Decrease Waiting Times in Emergency Department: Does it Make Sense?” in the autumn issue of Archives of Trauma Research (1). As an emergency physician in the United States, I can share the frustration of the authors that throughput of patients in the emergency department can often be hindered by numerous medical and logistical factors. In the United States, unlike most countries, emergency department care is not reserved to patients with acute severe conditions alone. Rather, under US law, emergency departments regularly provide care to patients who often do not have access to health care in other settings (2). I believe most emergency physicians in the United States would presume that the lack of such legal mandates would result in less crowding and difficulties in patient flow in emergency departments outside of the US. As this article clearly shows, unfortunately, there are common factors that emergency departments worldwide share in the challenge to improve patient waiting times. Similar to the US, the Iranian emergency department analyzed in this article faces obstacles in the form of personnel, lab processing and results and critical care bed availability. The authors chose a very appropriate simulation method to analyze how their emergency department staffing and procedures could be improved. I would be very curious to see in a follow-up article from the authors whether the implementation of potential solutions to the challenges identified was successful in reducing their emergency department waiting and patient holding times. Such solutions would be of great interest not only in Iranian emergency departments but also in emergency departments worldwide.
